# Asymmetric Preorganization of Inverted Pair Residues in the Sodium-Calcium Exchanger

**DOI:** 10.1038/srep20753

**Published:** 2016-02-15

**Authors:** Moshe Giladi, Lior Almagor, Liat van Dijk, Reuben Hiller, Petr Man, Eric Forest, Daniel Khananshvili

**Affiliations:** 1 Department of Physiology and Pharmacology, Sackler School of Medicine, Tel-Aviv University, Ramat-Aviv 69978, Israel; 2 Institute of Microbiology, Academy of Sciences of the Czech Republic, Prague, Czech Republic; 3 Univ. Grenoble Alpes, IBS, F-38044 Grenoble, France; 4 CNRS, IBS, F-38044 Grenoble, France; 5 CEA, IBS, F-38044 Grenoble, France

## Abstract

In analogy with many other proteins, Na^+^/Ca^2+^ exchangers (NCX) adapt an inverted twofold symmetry of repeated structural elements, while exhibiting a functional asymmetry by stabilizing an outward-facing conformation. Here, structure-based mutant analyses of the *Methanococcus jannaschii* Na^+^/Ca^2+^ exchanger (NCX_Mj) were performed in conjunction with HDX-MS (hydrogen/deuterium exchange mass spectrometry) to identify the structure-dynamic determinants of functional asymmetry. HDX-MS identified hallmark differences in backbone dynamics at ion-coordinating residues of apo-NCX_Mj, whereas Na^+^or Ca^2+^ binding to the respective sites induced relatively small, but specific, changes in backbone dynamics. Mutant analysis identified ion-coordinating residues affecting the catalytic capacity (*k*_cat_/K_m_), but not the stability of the outward-facing conformation. In contrast, distinct “noncatalytic” residues (adjacent to the ion-coordinating residues) control the stability of the outward-facing conformation, but not the catalytic capacity. The helix-breaking signature sequences (GTSLPE) on the α_1_ and α_2_ repeats (at the ion-binding core) differ in their folding/unfolding dynamics, while providing asymmetric contributions to transport activities. The present data strongly support the idea that asymmetric preorganization of the ligand-free ion-pocket predefines catalytic reorganization of ion-bound residues, where secondary interactions with adjacent residues couple the alternating access. These findings provide a structure-dynamic basis for ion-coupled alternating access in NCX and similar proteins.

Inverted twofold symmetry of repeated structural elements, which evolved via gene duplication and fusion events, represents a common motif among multi-helical proteins including enzymes, receptors, pumps, transporters, and channels. This structural set-up is particularly frequent in transporters^1^,^2^. This can be explained by the requirement to have isoenergetic alternating access, where the thermodynamic barrier can be lowered by “adjusting” the equilibrium constant close to unity^2^,^3^,^4^. This hypothetical set-up is especially appealing for symmetric small ligands, such as ions, although many transporters stabilize either the inward- or outward-facing state to fulfill physiological demands^3^,^4^,^5^,^6^,^7^,^8^. However, the underlying structure-dynamic mechanisms remain unclear.

Na^+^/Ca^2+^ exchanger (NCX) proteins extrude Ca^2+^ from the cell^5^,^6^ to control Ca^2+^ homeostasis^7^,^8^. NCX proteins catalyze the exchange (3Na^+^:1Ca^2+^)^9^, whereas the Na^+^ and Ca^2+^ ions are transported in separate steps^10^, consistent with the alternating access mechanism^11^,^12^. NCX orthologs vary as much as 10^4^-fold in the turnover rates of the transport cycle^13^,^14^,^15^,^16^ to match dynamic swings in cytosolic Ca^2+^ levels^7^,^8^. Despite these kinetic differences, NCXs share a common ability to stabilize the outward-facing (extracellular) access^13^,^17^. This is required to shape the physiological values of K_m_ at opposite sides of the membrane^7^,^8^,^13^,^17^. Although kinetics^16^,^17^ and structural studies^18^ revealed that under steady-state conditions the outward-facing conformation is stabilized in the presence of Na^+^ or Ca^2+^ ions, it has remained unclear whether the ligand-free NCX_Mj is already asymmetric in the absence of ions or ligand binding shifts the equilibrium in favor of the outward-facing conformation.

NCX proteins contain ten transmembrane helices that form two hubs (TM1-TM5 and TM6-TM10) with inverted twofold symmetry^1^,^2^,^5^,^6^,^7^,^8^. The crystal structure of ion-bound NCX_Mj depicts the outward-facing (extracellular) conformation (Fig. 1A) with four ion-binding sites, (S_ext_, S_mid_, S_int_, and S_Ca_), where twelve residues are involved in ion coordination^18^ (Fig. 1B). According to the structural data, the S_int_ and S_ext_ sites have high selectivity for Na^+^, whereas S_mid_ and S_Ca_ lack selectivity for ion binding. The ion pocket of NCX_Mj encompasses highly conserved α_1_ and α_2_ repeats with inverted topology, where twelve ion-coordinating residues (four in TM2 and TM7, and two in TM3 and TM8) and two helix-breaking signature sequences (GTSLPE) form the ion-pocket^18^,^19^,^20^,^21^ (Fig. 2A). According to the original interpretation of the crystallographic data, S_ext_, S_mid_, and S_int_ are occupied by 3Na^+^ ions, where E54, E213, D240, and N81 coordinate one Na^+^ ion at S_mid_, and 1Ca^2+^ occupies S_Ca_^18^. According to this interpretation, D240 and N81 solely belong to S_mid_ (Fig. 1B,D) and thus, are involved in Na^+^ binding/transport activities. Molecular dynamics simulations and ion-flux analyses offer an alternative interpretation, suggesting that 3Na^+^ ions occupy S_ext_, S_int_, and S_Ca_, whereas Ca^2+^ occupies S_Ca_^22^ (Fig. 1C,D). According to this point of view, S_mid_ does not bind either Na^+^ or Ca^2+^ ions and one water molecule is bound to protonated D240.

The present work was undertaken to evaluate the mechanistic role of inverted pair residues within the ion pocket of NCX_Mj by exploring especially suited kinetic analyses (Fig. 1E, S1,S2, and Table S1) of overexpressed NCX_Mj mutants possessing a right-side-out orientation^13^,^22^. In parallel, HDX MS (hydrogen/deuterium exchange mass spectrometry) experiments were performed on purified NCX_Mj proteins to monitor the backbone dynamics within the ion-binding pocket. In general, HDX-MS measures the exchange of backbone amide hydrogen with deuterium in solvent, where the measured HDX is related to the solvent’s accessibility and the backbone folding/unfolding dynamics (flexible regions take up more deuterium than do the rigid domains)^23^,^24^,^25^. Since HDX-MS can detect small and slow conformational changes in the presence or absence of ligand^26^,^27^, this technique is well suited for monitoring the folding/refolding dynamics of interest in NCX_Mj.

The present HDX-MS and kinetic studies revealed that the asymmetric preorganization of inverted pair residues in the ligand-free ion pocket predefines the catalytic reorganization of key ion-coordinating residues, where distinct “noncatalytic” residues stabilize the outward-facing conformation.

## Results

In order to envisage the backbone dynamics within the ion-binding pocket and assign the binding sites occupied by each ion, HDX-MS analysis^23^,^24^,^25^,^26^,^27^ was performed on apo and ion-bound NCX_Mj. This approach allows one to map not only the backbone dynamics in the absence of Na^+^ or Ca^2+^ —it also permits quantitative evaluation of local changes in backbone flexibility upon ion binding at respective sites. Highly purified preparations of NCX_Mj protein were used in all these experiments. MS identification of peptic peptides resulted in 26.7% coverage (Fig. S3), where three peptides cover the 41–59 area. Furthermore, two of them overlap, which led us to consider that three regions of rather small size (5–6 amides), namely, 41–46, 48–52, and 54–59 almost completely cover TM2. Eight peptides enabled us to cover the 195–264 area (from TM7 to the beginning of TM9). The peptides contain ten out of twelve ion-coordinating residues (except S77 and N81) and both α-repeats, including the GTSLPE signature sequence (Fig. 2A, S3, S4).

### HDX-MS reveals unusual HDX kinetics within the ion-binding pocket of NCX_Mj

The deuteration levels after 1,200 sec of exchange with and without ions are colored on the structure of NCX_Mj (Fig. 2B), and the corresponding deuterium uptake plots of each region or peptide at 15, 120, and 1,200 sec are shown in Fig. 2C. Interestingly, most peptides exhibited mixed EX1 and EX2 exchange kinetics of deuterium uptake. The EX1 kinetics exhibit hallmark bimodal isotope distribution, with the intensity of the one at high masses increasing with time relative to the one at low masses. EX1 kinetics implies cooperative unfolding events at specific segments of the ion-binding pocket and is quite unusual for conformational transitions that occur under physiologically related conditions (pH, ionic strength, temperature, among others)^28^. This indicates a local refolding rate slower than the labeling rate. For example, the presence of Ca^2+^ induced typical EX1 kinetics of HDX for peptide 215–230 (the end of TM7 and the beginning of TM8) (Fig. 2D). In contrast, the classical EX2 kinetics shows one binomial envelope moving towards higher masses with deuteration time^28^. A minor contribution of EX2 kinetics can also be detected because there is a slight shift towards higher masses in addition to the relative increase in the intensity of the high mass envelope (Fig. 2D).

### HDX-MS analysis of apo NCX_Mj protein

For Na^+^-free samples, Na^+^ was replaced by choline and for Ca^2+^-free samples, EDTA was added. Interestingly, in the absence of Na^+^ and Ca^2+^ ions, there are marked differences in the local backbone dynamics at key ion-coordinating residues in apo-NCX_Mj (Fig. 2B,C). For example, the 41–59 region, belonging to TM2 encompassing the α_1_-repeat, displayed distinct deuterium incorporations along the backbone of apo NCX. Overlapping peptides 47–59 and 53–59 enabled us to calculate a low deuterium uptake in region 48–52 (not more than 26% D), in contrast with a much higher deuterium uptake observed in region 54–59 (Fig. 2B,C). This dramatic change in deuterium uptake within TM2 may be induced by the tilting effect of P53, a phenomenon that has already been observed in transmembrane helices^29^. In sharp contrast with the 41–59 region, a pronounced deuterium uptake was measured in the 205–214 area of apo-NCX_Mj (Fig. 2B,C), which covers TM7 encompassing the α_2_-repeat. The significance of these findings is that in apo-NCX_Mj the backbone of the ion-coordinating residues, located on TM2 (S51 and E54), is much more rigid than the backbone of the ion-coordinating residues on TM7 (T209, E213, and D240).

### HDX-MS analysis of NCX_Mj protein in the presence of Na^+^ or Ca^2+^

**Figure 1:**
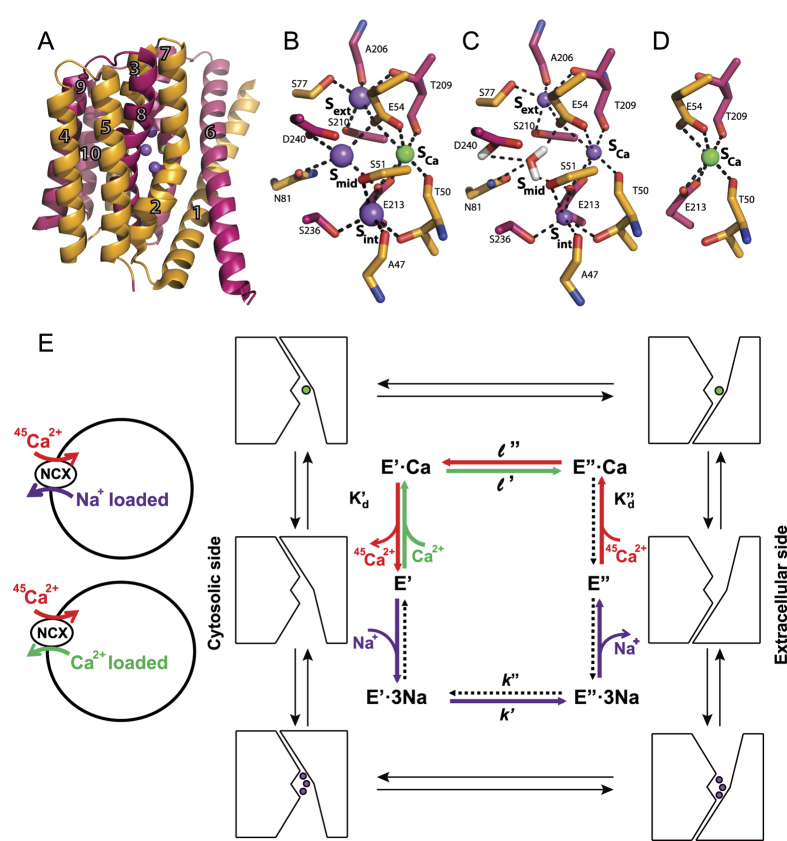
Structure and ion-exchange reactions of NCX_Mj. (**A**) Crystal structure of NCX_Mj (PDB 3V5U) in cartoon representation. Helices 1–5 (TM1-5) are orange and helices 6–10 (TM6-10) are pink. Purple and green spheres represent Na^+^ and Ca^2+^ ions, respectively. (**B**) Ion coordination as suggested by the crystal structure of NCX_Mj^18^. (**C**) 3Na^+^ ion coordination as suggested by molecular dynamics simulations and ion-flux assays^22^. (**D**) Ca^2+^ binding site. (**E**) Schematic representation of the ion-flux assay for Na^+^/^45^Ca^2+^ exchange or Ca^2+^/^45^Ca^2+^ exchange and the ping-pong mechanism describing the exchange reactions^10^,^16^,^17^. The red, green, and purple arrows represent Ca^2+^ -entry, Ca^2+^ -exit, and Na^+^ -exit steps of the transport cycle, respectively. The dotted arrows represent the reactions of the transport cycle, which negligibly contribute to the observed ion-exchange reactions under the given experimental conditions. The K′_d_ and K”_d_ values represent the dissociation constants for Ca^2+^ binding to NCX_Mj at the cytosolic and extracellular sides, respectively. The rate contributions to the observed *k*_cat_ and K_m_ values of the exchange reactions are expressed in the equations described in Table S1.

**Figure 2:**
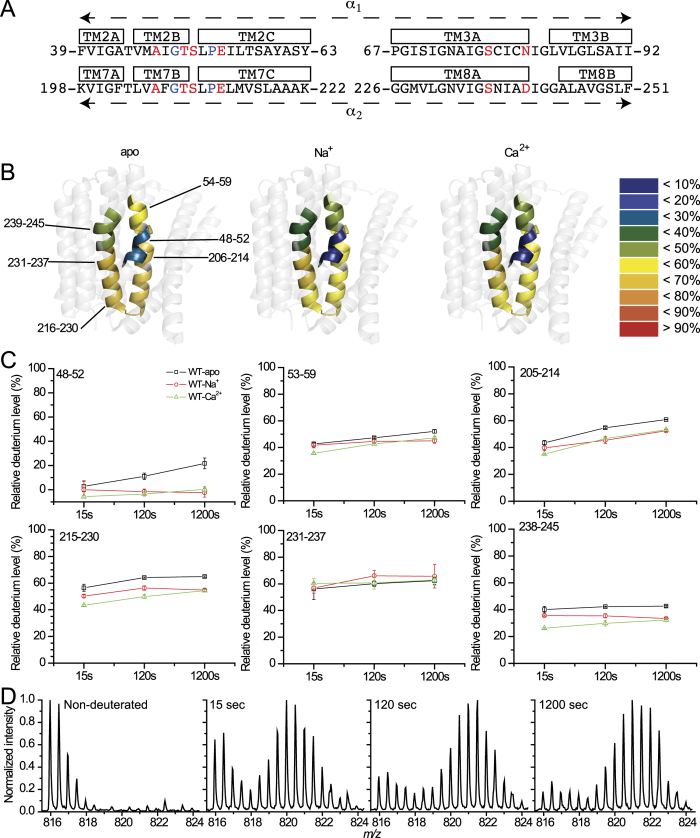
HDX-MS analysis of NCX_Mj. (**A**) Sequences of the α-repeats. Ion-coordinating residues are in red. Helix-breaking residues are in blue. (**B**) The heat map at 1,200 sec is overlaid on the crystal structure of NCX_Mj (PDB 3V5U) for the apo, Na^+^ -bound, and Ca^2+^ -bound forms. The color key indicates the HDX level. The numberings indicate regions with deuterium information (not taking into account the N-terminus amino acids of peptides without amide hydrogens). (**C**) Deuterium uptake plots for regions 48–52 and 231–237 and for peptides encompassing ion-coordinating residues. Data are presented as the mean ± SD (n = 3). Plots of regions 48–52 and 231–237 were calculated from the difference of deuteration between two overlapping peptides (47–59 & 53–59 and 215–230 & 215–237, respectively). (**D**) Mass spectra of peptide 215–230 in the presence of Ca^2+^. Note the characteristic EX1 kinetics bimodal isotope distribution, and the intensity of the one at high masses increasing with time relative to the one at a low masses.

The ion-bound species of NCX_Mj were studied using saturating concentrations for Na^+^ (100 mM) or Ca^2+^ (2 mM). In general, the exchange rate is higher for the apo form than for either the Na^+^ or Ca^2+^ bound forms (Fig. 2B,C). This is also true for peptide 215–230, which does not contain residues directly participating in ion ligation and, as mentioned, exhibits EX1 kinetics in the presence of Ca^2+^, indicating the local destabilization effect of Ca^2+^. This may be related to the ion-exchange mechanism. At 1,200 sec, the exchange of the relevant peptides (Fig. 2B,C) is similar for the Na^+^ and Ca^2+^ -bound forms. Interestingly, in the presence of Na^+^ or Ca^2+^, deuterium was not at all incorporated into the 48–52 region (no HDX in its five amides). This behavior may be related to the presence of Na^+^ ligands A47, T50, and S51 in S_int_ and the presence of Ca^2+^ ligands T50, S51, and E54 in S_Ca_, thus, stabilizing the backbone conformation in this region upon ion binding. Assuming the occupation of S_mid_ by Na^+ ^18 (Fig. 1B), one may expect that Na^+^ binding at S_mid_ will result in a major decrease in deuterium uptake for peptide 238–245 encompassing D240 (Fig. 2B,C); however, either Na^+^ or Ca^2+^ equally protects this region, as compared with the apo form. In fact, at 15 and 120 sec, Ca^2+^ protects this peptide more than Na^+^ does.

The two GTSLPE signature sequences on TM2 and TM7 (residues 49–54 and 208–213), although being “symmetry-related”, exhibit marked differences in HDX kinetics (Fig. 2B,C). The α_1_-repeat, at residues 49–54, exhibits relatively low deuterium uptake in both the apo and bound forms and is highly protected by the presence of either Na^+^ or Ca^2+^. In contrast, the α_2_-repeat at residues 208–213 (peptide 205–214) is much more dynamic in both the apo and bound forms and ligand binding results in less protection. This may represent the functional asymmetry of NCX_Mj^13^, implemented in an asymmetric arrangement of “catalytic residues” involved in transition state stabilization.

### Asymmetric contributions of inverted pair residues to ion-transport activities

**Figure 3:**
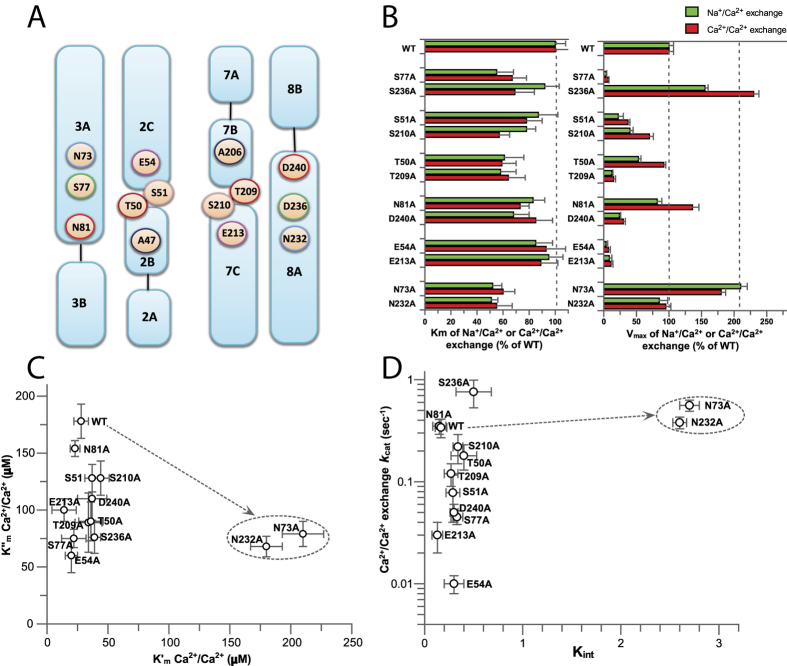
Mutational effects of pair residues on the intrinsic equilibrium (K_int_) of bidirectional Ca^2+^ movements (Ca^2+^/Ca^2+^ exchange). (**A**) Topological positions of pair residues within the ion pocket of NCX_Mj. Matching pair residues are marked with the same color. (**B**) The K_m_ and V_max_ values of the Na^+^/Ca^2+^ and Ca^2+^/Ca^2+^ exchange reactions were measured by varying the concentrations of ^45^Ca^2+^ (20–2000 μM) in the assay medium at fixed (saturating) concentrations of intravesicular Na^+^ (160 mM) or Ca^2+^ (250 μM), as described in Methods (see also Figure S1). The bars represent the mean ± SE. (**C**) The K_m_ values of the Ca^2+^/Ca^2+^ exchange reaction were measured at the ‘extracellular’ (K″_m_) and ‘cytosolic’ (K′_m_) sides by varying the Ca^2+^ concentrations in one compartment and keeping the fixed (saturating) concentrations of Ca^2+^ in the trans compartment. (**D**) The K_int_ values were calculated as the K′_m_/K″_m_ ratio and plotted *vs* the *k*_cat_ values for the same mutant. The *k*_cat_ values were calculated as *k*_cat_ = V_max_/[E]_t_. The expression levels of [E]_t_ were normalized by using the GFP assay (see Methods).

For structure-based mutational analyses of ion-transport mechanisms, the initial rates of Na^+^_i_ or Ca^2+^_i_-dependent ^45^Ca^2+^ -uptake were measured in right-side-oriented isolated vesicles derived from the plasma membranes of *E. coli* cells containing overexpressed NCX_Mj mutants^13^,^22^. In this experimental set-up the extracellular side matches the extravesicular orientation, where the kinetic parameters of the Na^+^/Ca^2+^ and Ca^2+^/Ca^2+^ exchange reactions at the ‘extracellular’ (K″_m_ and V″_max_) and ‘cytosolic’ (K′_m_ and V′_max_) sides can be measured by varying ion concentrations at the intravesicular and extravesicular compartments (Fig. 1E and Table S1). Single-point mutations to alanine were performed for ion-coordinating pair residues, namely, S77/S236, S51/S210, T50/T209, D240/N81, and E54/E213 (Fig. 3A). Unfortunately, the alanine-substitution approach cannot be applied for the A47/A206 pair^18^. The N73/N232 pair was also subjected to alanine substitutions. Although N73 and N232 do not directly coordinate the ions, they are located at inverted entries to the ion pocket (Fig. 3A) and form hydrogen bonding with T209 and T50, respectively.

In general, the tested mutations have rather minor effects on the apparent affinity of Ca^2+^ transport, exhibiting less than twofold changes in the K″_m_ values of the Na^+^/Ca^2+^ and Ca^2+^/Ca^2+^ exchange activities, whereas they have diverse effects on the V″_max_ values (Fig. 3B). The R_A_ index (representing the ratio of V″_max_ values for a given pair of pair residues) reveals the asymmetric effects of alanine substitutions on the ion exchange activities, with R_A_ values ranging from 2 to 32 (Table 1). Although the tested pair residues display R_A_ ≫ 1 values (reflecting their asymmetric contributions), the S77/S236 pair has an extraordinarily high R_A_ value for both the Na^+^/Ca^2+^ and Ca^2+^/Ca^2+^ exchanges, which is at least 10 times higher than the R_A_ value imposed by any other pair residues (Table 1).

### Mutational effects of ion pocket residues on the intrinsic equilibrium of bidirectional Ca^2+^ movements

Previous studies have shown that the K′_m_/K″_m_ ratio of the NCX-mediated Ca^2+^/Ca^2+^ exchange reaction accounts for the intrinsic equilibrium (K_int_) of bidirectional Ca^2+^ movements^16^,^17^. The K_int_ ≃ 0.15 observed for NCX_Mj represents the intrinsic asymmetry of bidirectional ion movements (see Table S1 and Fig. S2), suggesting that the rate of Ca^2+^ movement from the cytosolic to the extracellular space is 5–7 times faster than Ca^2+^ movement in the opposite direction^16^. According to this analytical approach, the plotting of K_int_ vs. *k*_cat_ values provides valuable information about mutational effects on rate-equilibrium relationships underlying the bidirectional Ca^2+^ movements. Thus, the K′_m_ and K″_m_ values of Ca^2+^/Ca^2+^ exchange were measured for each mutant (Fig. 3C) and the resulting K_int_ values were plotted against the *k*_cat_ values for the same mutant (Fig. 3D). A striking finding is that alanine substitutions of ion-coordinating residues have very little (if any) effect on the intrinsic asymmetry of bidirectional Ca^2+^ movements (K_int_ = 0.1–0.35), whereas the same mutations have rather dramatic effects on the turnover rates (*k*_cat_ = 0.01–0.8 s^−1^) of Ca^2+^/Ca^2+^ exchange (Fig. 3D). In sharp contrast, the alanine-substitutions of N73A or N232A increase the K_int_ value from 0.15 ± 0.05 to 2.8 ± 0.2, whereas they have relatively minor effects on the *k*_cat_ value (Fig. 3D). These data support the notion that the mutation of N73 or N232 stabilizes the inward-facing access conformation without appreciably affecting the catalytic capacity (*k*_cat_/K_m_). Collectively, the present data indicate that N73 and N232 control K_int_ by affecting the ground/occluded states of Ca^2+^ with very little (if any) effects on the transition state.

### Ion-coordinating residues stabilizing the transition state of Ca^2+^ transport

**Figure 4:**
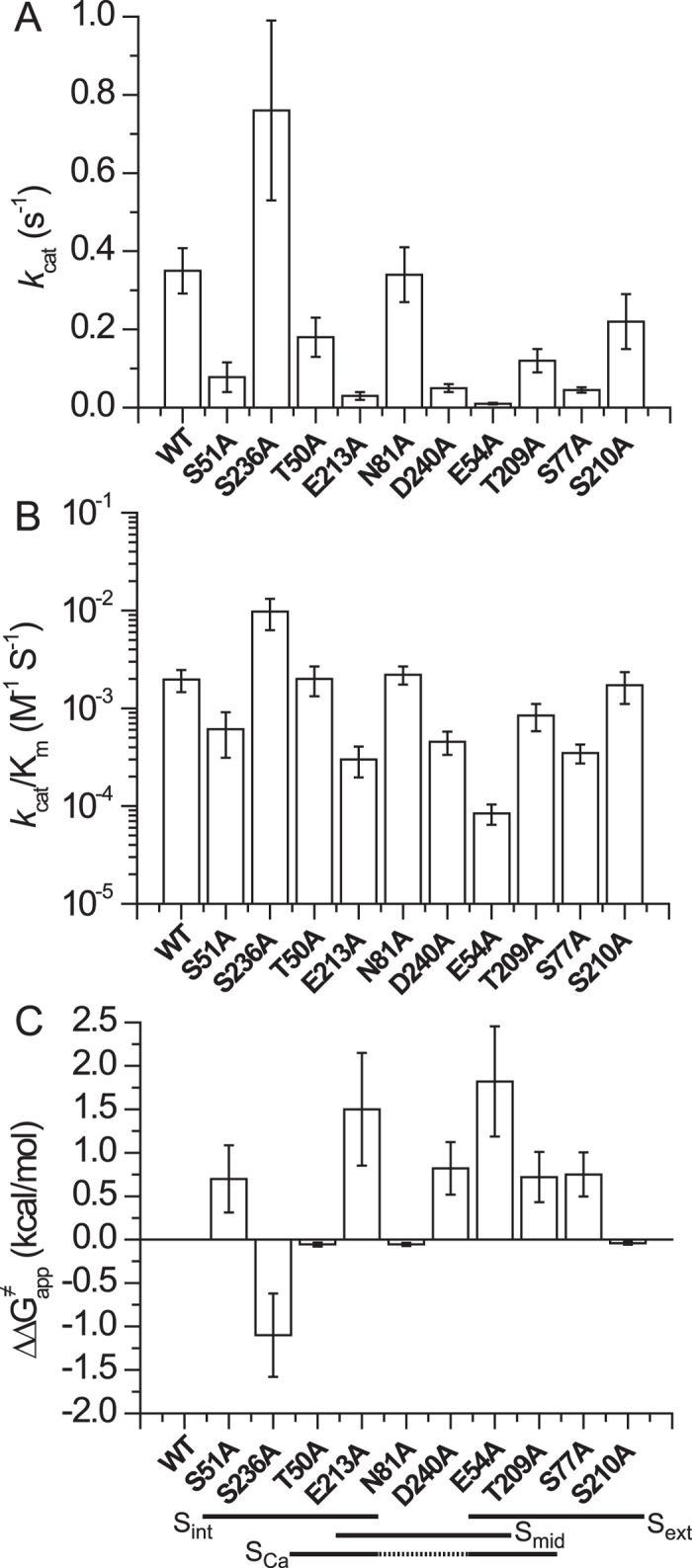
Identification of distinct residues stabilizing the Ca^2+^ transition state. (**A**) The K_m_ and V_max_ values of the Ca^2+^/Ca^2+^ exchange reaction were measured by varying the extravesicular Ca^2+^ concentrations (20–2000 μM) at saturating intravesicular Ca^2+^ (250 μM) (see Methods and Fig. S1). The *k*_cat_ values were calculated as described in the legend of Fig. 3D (see also Methods). The bars represent the mean ± SE. (**B**) The *k*_cat_/K_m_ values were derived from experimentally observed *k*_cat_ and K_m_ values, as outlined in panel **A**. The bars represent the mean ± SE. (**C**) Experimentally derived *k*_cat_ and K_m_ values of the Ca^2+^/Ca^2+^ exchange were used for calculating the ∆∆G^≠^_app_ values according to the equation ∆∆G^≠^_app_ = −2.303RT•log[(*k*_cat_/K_m_)_mut_/(*k*_cat_/K_m_)_wt_. The bars represent the mean ± SE.

Besides A47 and A206 (which coordinate Na^+^ at S_int_ and S_ext_, respectively, through their backbone carbonyls), all ion-coordinating side chains were replaced by alanine to evaluate the mutational effects on the *k*_cat_/K_m_ values of the Na^+^/Ca^2+^ or the Ca^2+^/Ca^2+^ exchange reactions (Figs 3 and 4). The K″_m_ and *k*_cat_ values of ^45^Ca^2+^ -uptake were measured by varying [^45^Ca^2+^]_o_ at saturating [Na^+^]_i_ or [Ca^2+^]_i_ (Fig. 1E, S1 and Table S1). E54A and E213A exhibit a 25–50-fold decrease in their *k*_cat_/K_m_ values (Figs 3B and 4A,B), whereas the polar (uncharged) residues, D240 (protonated, S_mid_), S51 (S_int_), T209 (S_ext_), and S77 (S_ext_) exhibit 10–25% of the WT *k*_cat_/K_m_ values for both exchange reactions (Figs 3B and 4A,B). The other polar residues (S236, S210, T50, and N81) have minor (if any) effects on either the *k*_cat_ or K_m_ values (Figs 3B and 4A,B). Interestingly, N81A retains the WT *k*_cat_ and K_m_ values for both ion-exchange reactions (Figs 3B and 4A).

**Figure 5:**
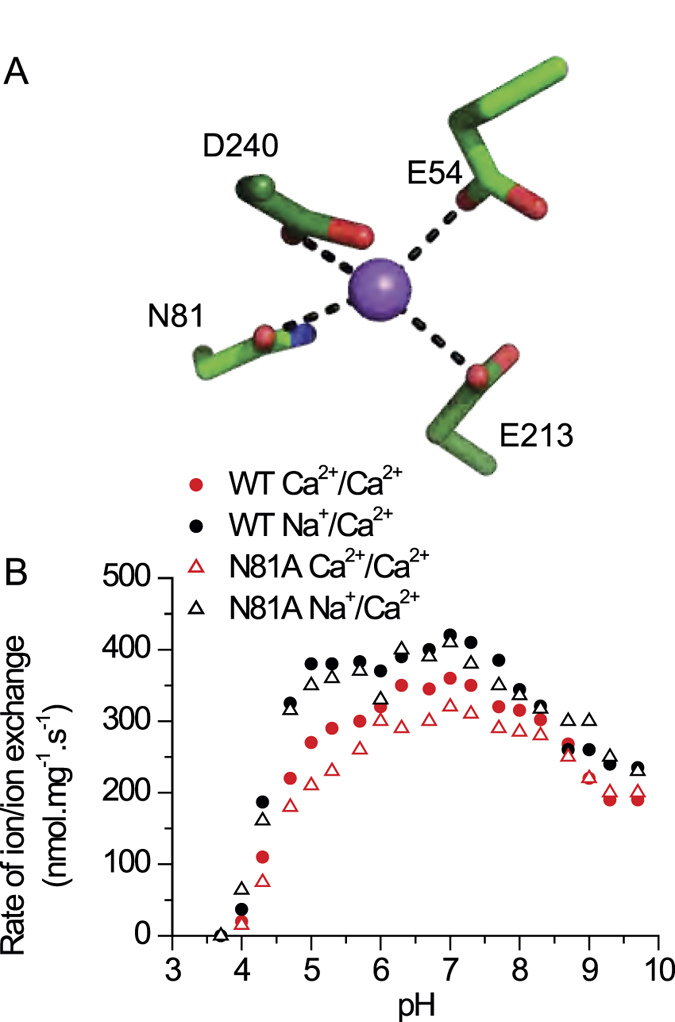
pH dependence of the ion-exchange reactions for the N81A mutant. (**A**) Na^+^ coordination at S_mid_, as originally suggested by the X-ray data of NCX_Mj. (**B**) The initial rates (t = 5 sec) of the Na^+^/Ca^2+^ and Ca^2+^/Ca^2+^ exchange reactions were measured as in the experiments shown in Figs 3 and 4, in an assay medium with 500 μM ^45^CaCl_2_. The intravesicular Na^+^ and Ca^2+^ values were 160 mM and 250 μM. The pH of the assay medium was controlled with a 20 mM MES/MOPS/Tris buffer.

According to the original interpretation of the crystallographic data, E54, E213, D240, and N81 coordinate one Na^+^ ion at the S_mid_ site, where D240 and N81 are mono-dentate residues that exclusively belong to the S_mid_ site^18^ (Fig. 5A). However, this interpretation has been recently challenged^22^ by molecular dynamics simulations and pH dependency analysis of exchange reactions in different mutants, although the N81 mutant was not tested in this study. To further assess the potential role of N81 ion binding/transport activities, the pH dependency of Ca^2+^/Ca^2+^ and Na^+^/Ca^2+^ exchanges were analyzed in N81A. The pH dependency curves of N81A for both ion-exchange reactions are very similar to those of WT NCX_Mj (Fig. 5B), suggesting that the side chain of N81 does not affect the ionization of neighboring catalytic residues, namely, E54, E213, and D240 (Fig. 4C). These results provide independent evidence for inability of the S_mid_ site to bind either Na^+^ nor Ca^2+^ in the ground state, in agreement with previous studies^22^.

To quantify the relative contributions of the ion-coordinating residues to transport catalysis, the observed ∆∆G^≠^_app_ values of the Ca^2+^/Ca^2+^ exchange reactions are presented for relevant mutants (∆∆G^≠^_app_ = −2.303RT•log[(*k*_cat_/K_m_)_mut_/(*k*_cat_/K_m_)_wt_]) (Fig. 4C). Notably, several ion-coordinating residues (S236, S210, T50, and N81) make negligible (if any) contributions to catalysis (∆∆G^≠^_app_ < 0.3 kcal/mol), whereas E54 and E213 are major residues that stabilize the transition-state, each contributing ∆∆G^≠^_app_ = 1.5–1.8 kcal/mol (Fig. 4C). Four ion-coordinating residues (D240, S51, S77, and T209) make moderate contributions to ion-transport activity (∆∆G^≠^_app_ = 0.7–0.8 kcal/mol), suggesting that these polar residues also participate in stabilizing the transition state. Thus, the side chains of two negatively charged residues (E54 and E213) and of four uncharged (polar) residues (protonated D240, S51, S77, and T209) stabilize the Ca^2+^ transition state (Fig. 4C).

### Proline/glycine residues of GTSLPE limit ion transport capacity

**Figure 6:**
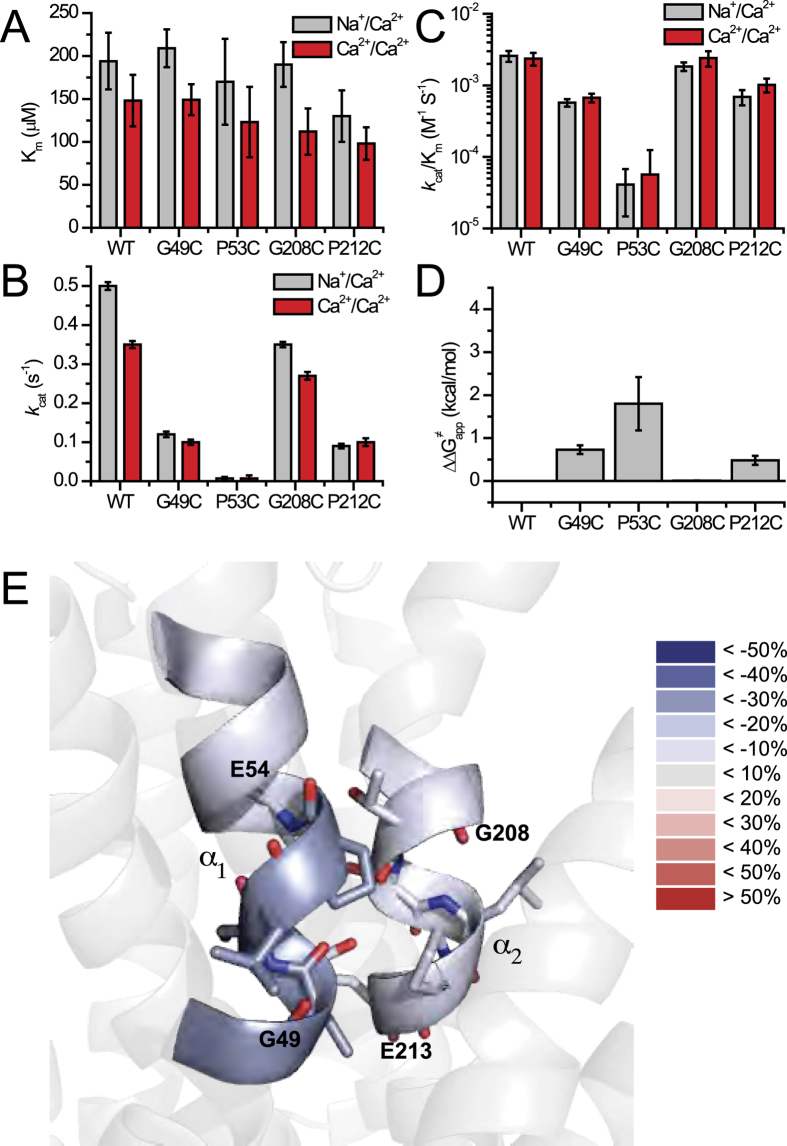
Mutational effects of GTSLPE proline and glycine on ion-exchange activities. (**A**) The K_m_ values of the Na^+^/Ca^2+^ and Ca^2+^/Ca^2+^ exchange reactions were measured by varying the concentrations of ^45^Ca^2+^ (20–2000 μM) in the assay medium and using saturating concentrations of intravesicular Na^+^ (160 mM) or Ca^2+^ (250 μM), as described in Methods (see also Fig. S1). The bars represent the mean ± SE. (**B**) The *k*_cat_ values were calculated as *k*_cat_ = V_max_/[E]_t_, where [E]_t_ was normalized by using the GFP assay. The bars represent the mean ± SE. (**C**) The *k*_cat_/K_m_ values were derived from experimentally observed *k*_cat_ and K_m_ values as outlined in panels (**A)** and (**B**). The bars represent the mean ± SE. (**D**) Experimentally derived values of *k*_cat_ and K_m_ of the Ca^2+^/Ca^2+^ exchange reaction were used for calculating the ∆∆G^≠^_app_ values according to the equation ∆∆G^≠^_app_ = −2.303RT•log[(*k*_cat_/K_m_)_mut_/(*k*_cat_/K_m_)_wt_. The bars represent the mean ± SE. (**E**) The difference between the HDX profile of the apo and Na^+^-bound forms of NCX_Mj at 1200 sec are overlaid on the crystal structure of NCX_Mj (PDB 3V5U). The color key indicates the difference in the percentage of deuterium incorporation between the Na^+^-bound and apo forms. The blue labeling corresponds to regions with less deuterium incorporation in the Na^+^-bound form compared with the apo form. The results are nearly identical for the Ca^2+^- and Na^+^-bound species.

The glycine and proline residues in the signature sequences 49-GTSLPE-54 (TM2) and 208-GTSLPE-213 (TM7) are not directly involved in Na^+^ or Ca^2+^ coordination, although these residues are highly conserved among NCXs and other membrane proteins^18^,^30^. This is especially interesting in light of the fact that G49, G208, P53, and P212 are located near the key functional residues E54 and E213. In general, mutations of glycine and proline residues have no appreciable effect on the K_m_ values of either ion-exchange reaction (Fig. 6A). Although G208C largely retains the WT properties, G49C exhibits ~10-fold decrease in the *k*_cat_/K_m_ values (Fig. 6B,C). Thus, G49C and G208C have different effects on ion transport activities, although these two glycine residues represent symmetrically related positions in the inverted α_1_/α_2_ repeats of NCX_Mj. This finding is in agreement with the different levels of HDX (Fig. 2B,C) and ligand protection at the signature GTSLPE repeats on TM2 and TM7 (Fig. 6E). Interestingly, P53C and P212C retain only ~3% and ~12% of the WT *k*_cat_/K_m_ values, respectively (Fig. 6B,C), thereby suggesting that both proline residues may restrict the backbone flexibility within the ion-binding pocket, consequently affecting the ion transport activities (Fig. 6D). Although G208C does not affect the ion transport activities, the other three helix-breaking residues (G49, P53, and P212) are actively involved in ion transport activities with ∆∆G^≠^_app_ = 0.5–1.8 kcal/mol (Fig. 6D,E). This suggests that the structure-encoded energy of backbone folding/unfolding at critical residues controls the transition state’s stabilization.

## Discussion

The present work was undertaken to analyze the structure-dynamic determinants of ion-transport catalysis and the intrinsic asymmetry of bidirectional movements in the NCX_Mj protein as related to the inverted twofold symmetry of ion-coordinating pair residues. For this purpose, structure-based kinetic analyses of mutants were performed by testing the effect of alanine mutations on the catalytic capacity (*k*_cat_ /K_m_) and on the intrinsic equilibrium of bidirectional Ca^2+^ movements (K_int_ = K′_m_/K″_m_) by assaying the Ca^2+^/Ca^2+^ exchange reaction in *E. coli*-derived cell-membrane vesicles containing overexpressed NCX_Mj proteins with a right-side-out orientation. In another set of experiments the backbone dynamics of purified NCX_Mj were investigated in the presence or absence of Na^+^ or Ca^2+^ ions using HDX-MS, with the goal of tracking the protein backbone dynamics in the ligand-free and ion-bound pocket of NCX_Mj.

In general, HDX-MS is the technique of choice for investigating the structure-dynamic features of local backbone dynamics in apo- and ligand-bound proteins, although the outcome of valuable information depends very much on the sequence coverage in the mass spectrometry experiments. The observed sequence coverage (26.7%) of the NCX_Mj protein is quite low (Fig. S3) compared with that obtained with soluble proteins, mainly due to the detergent that often protects the protein from the protease under conditions suitable for HDX (quick digestions at low pHs and low temperatures). However, the regions of interest (TM2, TM7, and TM8), containing ten out of twelve ion-coordinating residues (except S77 and N81) on the α_1_ and α_2_ repeats, are well covered in the HDX-MS experiments. Notably, TM4, TM5, TM6, TM9, and TM10 neither contain any ion-binding residues nor are expected to undergo any significant conformational changes during the alternative access. Thus, the missing information from these sites is less relevant within the scope of the present work. It is somewhat regrettable that the present HDX-MS studies did not cover the TM1/TM6 segments, since these helices may represent the moving gating bundle associated with alternative access. However, this drawback seems to be tolerable as well, since the scope of the present work does not focus on the ion-coupled “sliding” of the gating bundle. Although the present studies provide a clue for identifying the structure-dynamic determinants controlling the ion-coupled sliding of the gating bundle (see below), more dedicated research is required for elucidating the relevant mechanism.

The HDX-MS experiments described here demonstrate that the “symmetric” repeating structures exhibit inherently asymmetrical dynamics, observed in the absence of Na^+^ or Ca^2+^ (Fig. 2). These variances in the pattern of backbone dynamics include the strictly conserved α-repeats within the ion-binding pocket, where the local backbone dynamics are much more constrained at T50 and S51 (both of these residues belong to the 48–52 region showing low HDX) (Fig. 2B,C). This implies a unique pattern of dynamic preorganization of catalytic residues in the absence of a bound ligand. Moreover, three helix-breaking residues (G49, P53, and P212), located nearby E54 and E213, actively contribute to the helix folding/refolding dynamics at critical residues, thereby limiting their ion-transport activities (Fig. 6). Notably, G208 is located on a very flexible segment of TM7B (Fig. 2), which may explain why the mutation of this residue does not limit ion transport activities in NCX_Mj. These findings, in conjunction with recent findings^27^, raise the possibility that the ion-binding pocket of mammalian NCX1 is more flexible than that of NCX_Mj. The reason for this could lie in the structure-dynamic and functional differences in the helix-breaking signature sequences as well as the length of helices involved in ion coordination.

Next, we examined how ligand binding affects the structure dynamics. The data revealed a fairly moderate effect of ion binding on the HDX profiles (Fig. 2). However, these changes are highly specific to previously resolved binding sites. Notably, the strength and location of Na^+^ and Ca^2+^-dependent effects on deuterium uptake are comparable, thereby suggesting that both ions rigidify local backbone dynamics similarly (Fig. 2). The emergence of EX1 kinetics upon ligand binding (Fig. 2D) is intriguing. It is tempting to speculate that upon ligand binding, alternative access proceeds through multiple local unfolding/refolding steps requiring low activation energies rather than through a few large, concerted conformational changes, requiring high activation energies.

Alanine substitutions have identified three different groups of residues having diverse effects on the catalytic capacity (*k*_cat_/K_m_) and intrinsic equilibrium (K_int_) of bidirectional Ca^2+^-movements (Figs 3 and 4). In the first group, the alanine mutations of four ion-coordinating residues (S236, S210, T50, and N81) have no appreciable effects either on the *k*_cat_/K_m_ or K_int_ values, thereby suggesting that these residues neither control the ion-transport catalysis nor the intrinsic equilibrium of bidirectional ion movements. In the second group of residues (S51, E54, S77, T209, E213, and D240) the alanine substitutions affect the *k*_cat_/K_m_ but not the K_int_ value (Figs 3 and 4B). Thus, these residues control ion-transport catalysis without affecting the intrinsic equilibrium of bidirectional ion movements. In the third group of residues (N73 and N232), the alanine mutations have no appreciable effects on the k_cat_/K_m_ values, but result in up to 20-fold changes in K_int_, thereby revealing inward-facing conformation stabilization (K_int_ = 2–3) in these mutants (Fig. 3D).

**Figure 7:**
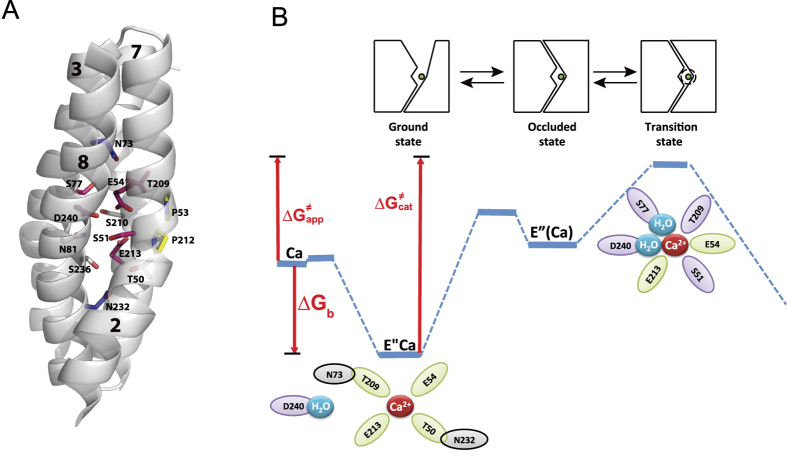
Proposed mechanism underlying the catalytic preorganization for Ca^2+^ -bound species in the NCX_Mj antiporter. (**A**) Inverted pair residues (shown in matching colors) contributing to the ion-transport activities, bidirectional ion movements, and local backbone dynamics at key functional residues are represented as sticks. (**B**) On the basis of the available experimental data, it is proposed that the backbone carbonyls of T209 and T50 and side chains of E54 and E213 bind Ca^2+^ in the ground state, where N73 and N232 form a hydrogen bond with T209 and T50, respectively (Fig. 1D). According to the available data, the side chains of E54, E213, D240, S51, T209, and S77 stabilize Ca^2+^ in the transition state, where ∆G_b_ = RTlnK_m_; ∆G^≠^_app_ = −RTln(*k*_cat_/K_m_); ∆G^≠^_cat_ = −RTln*k*_cat_.

Although N73 and N232 are not directly involved in ion coordination, according to the crystal structure, N73 and N232 can form a hydrogen bond with T209 and T50, respectively. Since the T209 and T50 side chains contribute to the Na^+^ sites, whereas their backbone carbonyls form the Ca^2+^ site (Figs 1C,D and 7A), the N73A and N232A mutations may affect the stability of the Ca^2+^ site and thus, may affect the asymmetry of bidirectional ion movements (Fig. 7A). The mechanistic significance of this rationale is that N73 and N232 may control one or more occluded states of ion-bound species, while favorably stabilizing the outward-facing conformation during alternating access. Therefore, N73 and N232 may play a critical role in coupling the conformational rearrangement of cytosolic and extracellular gates upon Ca^2+^ or Na^+^ binding.

In general, the observed ∆∆G^≠^_app_ values (0.5–1.8 kcal/mol) for “catalytic residues” (Fig. 4D), based on the *k*_cat_/K_m_ measurements, are too small to result in large conformational changes in the local backbone dynamics of the ion-binding pocket. This is in good agreement with the observed small changes in deuterium uptake within the ion-binding pocket upon Ca^2+^ or Na^+^ binding, observed in the HDX-MS experiments (Figs 2, 3, and S4). Notably, E54 and E213 make the most prominent contributions to transition-state stabilization (∆∆G^≠^_app_ = 1.5–1.8 kcal/mol), in conjunction with four polar residues, namely, S51, S77, T209, and D240 (Fig. 4D). These findings suggest that a polar-electrostatic mechanism governs catalytic reorganization^31^,^32^, involving the asymmetric contribution of inverted pair residues to the stabilization of the transition state (Fig. 4A,D and Fig. 7). Notably, the sum of the observed ∆∆G^≠^_app_ values of “catalytic” ion-coordinating residues adds up to ~5.8 kcal/mol (Figs 4C and 6D), which approaches the experimentally derived value of ∆G^≠^_app_ = 4.9 kcal for WT NCX_Mj (Fig. 7). Careful computational studies are required for evaluating the physical meaning of the observed ∆∆G^≠^_app_ values in relation to ∆G^≠^_app_ and ∆G^≠^_cat_. Although the exact mechanism underlying ion-coupled alternative access remains to be resolved, the present findings are consistent with the notion that asymmetric preorganization of key residues in apo-NCX_Mj predefines the differential contributions of matching pair residues toward ion occlusion and the reoganization of catalytic residues (Fig. 7B). This information may provide a structure-dynamic basis for elucidating the exact mechanisms underlying ion-coupled alternating access in NCX and similar proteins, since the present findings provide a clue for determining the future experimental design and impose physical restrictions for computational studies.

Collectively, our data support the notion that the α_1_-repeat (at TM2) serves as a rigid anchor around which the catalytic residues E54 (S_Ca_), E213 (S_Ca_), protonated D240 (S_mid_), S51 (S_int_), S77 (S_ext_), and T209 (S_ext_) stabilize the transition state (Figs 4C, 7, and S4). Therefore, the six catalytic residues belonging to all four ion-binding sites take part in the transition state (Fig. 7B), whereas in the ground state only the S_Ca_ site is involved in Ca^2+^ binding (Fig. 1C,D). The helix-breaking signature sequences (GTSLPE) on α_1_ and α_2_ also contribute to transition-state stabilization, presumably because they lopsidedly restrict the backbone dynamics within the ion-binding pocket and thereby, pre-establish the dynamic asymmetry in the ligand-free protein (Figs 1 and 6). Collectively, Ca^2+^ ligation by “rigid residues” (E54 and S51) may largely predefine the positioning of catalytic residues in the transition state (thereby governing the asymmetric nature of catalytic reorganization), whereas the other catalytic residues (E213, D240, T209, and S77) may possess a higher degree of freedom for Ca^2+^ interaction in the transition state (Figs 2 and 7). Based on stereochemical considerations, it is tempting to speculate that two water molecules may contribute to the stabilization of the Ca^2+^ transition state through S77 and/or D240 (Fig. 7).

In conclusion, we found hallmark differences in the local backbone dynamics at key ion-coordinating residues within the ligand-free ion-pocket of apo-NCX_Mj, whereas Na^+^ or Ca^2+^ binding to the respective sites induced relatively small, but specific changes in the backbone dynamics. In addition, the catalytic capacity and the intrinsic equilibrium of bidirectional ion movements are controlled by asymmetric contributions of inverted pair residues, thereby revealing a specific preorganization of ion-coordinating and helix-breaking residues within the ion-binding pocket. Thus, inverted structural elements in NCX_Mj are asymmetrically preorganized at the single-residue level, thus providing a structure-dynamic basis for asymmetric contributions to the catalytic reorganization. The currently revealed asymmetric nature of ligand-free preorganization and the catalytic reorganization may provide a clue for assessing the mechanism underlying ion-coupled alternating access. Taking into account the structural similarities of NCX_Mj to other proteins belonging to the superfamily of Ca^2+^/CA antiporters^7^,^8^,^18^,^19^,^20^,^22^, the present findings may have general significance for attaining a better understanding of the mechanisms underlying alternating access and transport catalysis in secondary transport systems.

## Methods

### Construct preparation

DNA encoding the WT NCX_Mj was amplified by PCR from a *Methanocaldococcus jannaschii* cDNA library (DSMZ) and ligated between the NcoI and BamHI restriction sites of a pET-28a plasmid^13^,^22^. Mutations were introduced by QuickChange mutagenesis (Stratagene) and were confirmed by sequencing.

### *E. coli*-derived vesicles containing overexpressed NCX_Mj mutants

Expression vectors were transformed into *E. coli* BL21 (DE3) pLysS competent cells. Cells were grown, harvested, homogenized, and lysed by French-Press^13^,^22^. Cell lysates were loaded onto a three-step sucrose gradient and membrane vesicles were stored in 5 mM Mops-Tris pH 7.4, 250 mM sucrose at −80 °C until use^13^,^17^. The expression levels of NCX_Mj were evaluated using the GFP-assay. The orientation of NCX_Mj in *E. coli*-derived cell-membrane vesicles was tested using an antibody against the 6xHis-tag^13^.

### GFP-assay for evaluating the expression levels of NCX_Mj

The ratio of NCX-Mj expression to the total vesicle protein was approximated by calculating the concentration of GFP by its absorbance at 488 nm (e.c. = 56000) and dividing it by the value determined by the Lowry assay. The GFP absorbance signal at 488 nm was isolated from vesicle noise using a correction factor determined by measuring non-expressing vesicles^13^.

### Protein purification

NCX_Mj was overexpressed and purified as outlined before^13^,^18^,^33^. Briefly, membranes were isolated from the cell lysate by ultracentrifugation and after membrane protein extraction with 20 mM DDM (n-Dodecyl β-D-maltoside), the supernatant was loaded onto a Talon Co^2+^ affinity column. Protein was then desalted to eliminate imidazole with a buffer containing 2 mM DDM and digested overnight with TEV protease. Following a second passage through the Co^2+^ column (with a buffer containing 2 mM DDM) to eliminate the His-tag and TEV protease, the protein was purified on Superdex-200 pre-equilibrated with 0.5 mM DDM buffer. Concentrated (1–2.5 mg/ml) preparations of purified proteins (>95% purity, judged by SDS-PAGE) were stored at −80 °C in buffer containing 0.5 mM DDM.

### ^45^Ca^2+^-uptake assay in *E. coli*-derived vesicles

The initial rates of the Na^+^/Ca^2+^ and Ca^2+^/Ca^2+^ exchange reactions were assayed by measuring ^45^Ca^2+^-uptake in the *E. coli*-derived vesicles containing the overexpressed WT or mutants of NCX_Mj, as previously described^13^,^17^,^22^. Briefly, Na^+^ (160 mM) or Ca^2+^ (250 μM)-loaded vesicles were rapidly diluted 25–50-fold at 35 °C in an assay medium containing 20 mM MOPS/Tris, pH 6.5, 100 mM KCl, and 5–2000 μM ^45^CaCl_2_. The ^45^Ca^2+^-uptake was quenched by cold buffer containing 10 mM EGTA and filtrated on GF/C filters, as outlined before^13^,^17^,^22^. The K_m_ and V_max_ values were measured in at least three independent experiments (data are presented as mean ± SEM) with GraFit 7.1 (Erithacus Software, Ltd.)^10^,^13^,^17^. The *k*_cat_ was calculated as *k*_cat_ = V_max_/[E]_t_, where [E]_t_ was determined by the GFP assay (see above).

### Peptide analysis by tandem mass spectrometry (MS/MS) after NCX_Mj proteolysis

LC-MS/MS analyses were performed under H/D exchange compatible conditions and data analysis was performed with MStools^34^. The system consisted of injection and switching valves mounted with a desalting cartridge (peptide Opti-Trap Micro from Optimize Technologies) and an analytical column (Jupiter C18, 0.5 × 50 mm, 5 μm, 300 Å, Phenomenex). NCX_Mj was digested using a porcine pepsin (Sigma) solution at 0.23 mg/mL in 100 mM glycine-HCl, pH 2.5 in an ice-water bath for 2 min. Desalting (3 min) was driven by a Shimadzu LC20-AD pump isocratically (0.4% formic acid in water at 100 μL/min). Gradient was separated using the HPLC system (Agilent Technologies 1200) at a flow rate of 15 μL/min. Gradient elution (from 10% B to 40% B in 25 min), followed by 1 min gradient to 95% B, was used for separation. The solvents used were A–0.4% formic acid, 2% acetonitrile in water, and solvent B–0.4% formic acid in 95% acetonitrile. The outlet of the analytical column was directly connected to an electrospray ionization (ESI) source of an Apex-ULTRA Qe Fourier transform ion cyclotron resonance (FT-ICR) mass spectrometer (Bruker Daltonics) equipped with a 9.4 T superconducting magnet. ESI-FT-ICR MS was calibrated externally using arginine clusters, resulting in a mass accuracy below 2 ppm. For LC-MS/MS the instrument was operated in data-dependent mode by selecting the six most intense ions in each MS scan for an MS/MS analysis using collisionally induced dissociation in a quadrupole. The data were searched by MASCOT (MatrixScience) against single-protein databases containing the NCX sequence and against a database containing pepsin (to exclude possible autolysis products).

### Hydrogen/Deuterium exchange Mass Spectrometry (HDX-MS)

HDX-MS experiments were fully automated using a PAL autosampler (CTC Analytics). This controlled the start of the exchange and quench reactions, the proteolysis temperature (4 °C), the injection of the deuterated peptides, as well as management of the injection and washing valves; it also triggered the acquisition of the mass spectrometer and HPLC pumps. A Peltier-cooled box (4 °C) contained two Rheodyne automated valves, a desalting cartridge (peptide Opti-Trap Micro from Optimize Technologies) and a HPLC column (Jupiter 4μm Proteo, Phenomenex). HDX MS reactions were carried out using different forms of WT NCX_Mj (apo, with Na^+^ or Ca^2+^) at a concentration of 40 μM. Deuteration was initiated by a 5-fold dilution of the protein samples (10 μL) with the same buffer in D_2_O (40 μL). The proteins were deuterated for 15 sec, or for 2 or 20 min at 4 °C. Back-exchange quench and digestion were performed using 50 μL of porcine pepsin (Sigma) solution at 0.23 mg/mL in 100 mM glycine-HCl, pH 2.5 at 4 °C for 2 min. The peptides were desalted for 6 min using a HPLC pump (Agilent Technologies) with 0.03% TFA in water, at a flow rate of 100 μL/min. The peptides were separated using another HPLC pump (Agilent Technologies) at 50 μL/min for 6 min with 15–50% gradient B (Buffer A: TFA 0.03% in water; Buffer B: acetonitrile 95%, TFA 0.03% in water), followed by 9 min at 50% B and 1 min at 100% B. The peptide masses were measured using an electrospray-TOF mass spectrometer (Agilent 6210) in the 300–1300 m/z range. Each deuteration experiment was conducted in triplicate. The Mass Hunter (Agilent Technologies) software was used for data acquisition. The HD Examiner software (Sierra Analytics) was used for HDX-MS data processing. MSTools was used for visualization and presentation of the HDX-MS data^34^.

## Additional Information

**How to cite this article**: Giladi, M. *et al.* Asymmetric Preorganization of Inverted Pair Residues in the Sodium-Calcium Exchanger. *Sci. Rep.* **6**, 20753; doi: 10.1038/srep20753 (2016).

## Supplementary Material

Supplementary Information

## Figures and Tables

**Table 1:** Relative effects of alanine mutations on the ion-exchange capacity in a given pair of inverted residues.

Mutated pair residues	R_A_ values (V″_max1_/V″_max2_) Na/Ca exchange	R_A_ values (V″_max1_/V″_max2_) Ca/Ca exchange
S236A/S77A	31.3 ± 3.6	32.8 ± 2.3
N81A/D240A	3.5 ± 0.4	4.5 ± 0.3
T50A/T209A	2.5 ± 0.3	3.0 ± 0.3
S210A/S51A	2.2 ± 0.3	1.9 ± 0.3
E213A/E54A	1.8 ± 0.4	1.6 ± 0.3
N73A/N232A	2.1 ± 0.3	1.7 ± 0.3

The V″_max1_ and V″_max2_ ratios of the Na^+^/Ca^2+^ and Ca^2+^/Ca^2+^ exchange reactions were measured in mutants of matching pair residues as described in Figs 3 and 4 (see also Methods). The V″_max1_/V″_max2_ ratios are presented as the index of asymmetry (R_A_) for a given pair of residues. The numbers represent the mean ± SE.
