# Supplementary material for: Asymmetric Preorganization of Inverted Pair Residues in the Sodium-Calcium Exchanger
Source: Sci Rep. 2016 Feb 15;6:20753. doi: 10.1038/srep20753 (PMC4753433; doi:10.1038/srep20753)
Supplement: Supplementary Information [file srep20753-s1.pdf]

Supporting information for

Asymmetric Preorganization of Inverted Pair Residues in the Sodium-Calcium Exchanger

Moshe Giladi[a,1], Lior Almagor[a,1], Liat van Dijk[a], Reuben Hiller[a], Petr Man[b], Eric Forest[c,d,e*] and Daniel Khananshvili[a*]

[a]Department of Physiology and Pharmacology, Sackler School of Medicine, Tel-Aviv University, Ramat-Aviv 69978, Israel.
[b]Institute of Microbiology, Academy of Sciences of the Czech Republic, Prague, Czech Republic.
[c]Univ. Grenoble Alpes, IBS, F-38044 Grenoble, France
[d]CNRS, IBS, F-38044 Grenoble, France
[e]CEA, IBS, F-38044 Grenoble, France

[1] These authors contributed equally

***Corresponding authors**:

*Dr. Eric Forest, Institut de Biologie Structurale,
EPN Campus, 71 avenue des Martyrs
CS 10090, 38044 Grenoble Cedex 9, France
E-mail: eric.forest@ibs.fr

*Prof. Daniel Khananshvili
Department of Physiology and Pharmacology
Sackler School of Medicine, Tel-Aviv University
Ramat-Aviv, Tel-Aviv 69978, Israel
Tel: 972-3-640-9961; Fax: 972-3-640-9113
E-mail: dhanan@post.tau.ac.il

This file includes:

Figure S1 to S4;

Table S1

FIGURE S1. $^{45}Ca^{2+}$-flux assays of Na^+/Ca^{2+} and Ca^{2+}/Ca^{2+} exchange reactions. (A) The time course of Na^+_i-dependent $^{45}Ca^{2+}$-uptake was measured in vesicles containing either overexpressed WT or mutated NCX_Mj. The Na^+-loaded (160 mM) vesicles were rapidly diluted (20-50-fold) in the assay medium containing 20 mM Mops/Tris, pH 6.5, 100 mM KCl, and 200 µM $^{45}CaCl_2$ at 35°C and the Ca^{2+}-uptake was stopped at the indicated times by injecting a cold EGTA-containing buffer. Quenched solutions were filtered and the radioactivity retained on the filters was assayed using a scintillation solution. **(B)** The initial rates (t = 5 s) of the Na^+/Ca^{2+} and Ca^{2+}/Ca^{2+} exchange reactions were measured by diluting the Na^+-loaded (160 mM) or Ca^{2+}-loaded (250 µM) vesicles in an assay medium containing 20-2000 µM $^{45}CaCl_2$. The exchange reactions were stopped and $^{45}Ca^{2+}$-uptake was measured as described above. **(C)** The equilibrium binding of $^{45}Ca^{2+}$ to purified WT NCX_Mj was measured by using the protein ultrafiltration method[1,2]. Briefly, an assay medium containing 30-50 µM protein with 100 mM KCl and 10 mM Tris-HCl at pH 7.2 was placed in the upper chamber of an Ultracel-3k concentrator and the assay was performed as previously outlined[1,2]. The concentrations of residual $[Ca^{2+}]_{res}$ in the decalcified assay buffers were measured by using the Fluo-3 assay[2]. The $^{45}Ca^{2+}$-binding titration curves were fit using GraFit software v7.1 (Erithacus Software, Ltd). The K_d value was derived by fitting the experimental points obtained by $^{45}Ca^{2+}$-titration curves.

FIGURE S2. **Quantitative assay of the intrinsic equilibrium (K_{int}) of bidirectional Ca^{2+}movements –** According to the ping-pong mechanism, describing the unidirectional Na^+/Ca^{2+} exchange cycle or the bidirectional Ca^{2+}/Ca^{2+} exchange (Figure 1E), the "apparent binding affinity" of Ca^{2+} (K''_m or K'_m) at opposite sides of the membrane can be

represented as a function of the intrinsic binding constant (K''_{Ca}, K'_{Ca}) and of the ion-translocation rate constants (*l',l",k',k"*) (Table S1). According to this formalism, the K'_m/K''_m ratio of the Ca^{2+}/Ca^{2+} exchange represents the intrinsic equilibrium (K_{int}) for bidirectional Ca^{2+} movements (Equation 1), which is related to Ca^{2+} binding at opposite sides of the membrane[4,5].

$$K_{int} = \frac{K'_m(Ca/Ca)}{K''_m(Ca/Ca)} = \frac{K'_{Ca}}{K''_{Ca}}\frac{l''}{l'} \quad (Equation\ 1)$$

According to this formalism, the experimentally measured values of cytosolic K'_m =28.2±3.5 µM and extracellular K''_m = 183.9±27.7 µM of the Ca^{2+}/Ca^{2+} exchange (Figure 3B) result in a K_{int} value of 0.15±0.05 (n=5), thereby revealing an intrinsic asymmetry of bidirectional Ca^{2+} movements favoring the extracellular access. By assuming that Ca^{2+} binding site of NCX_Mj (S_{Ca}) has a comparable intrinsic affinity for Ca^{2+} binding ($K'_{Ca} \approx K''_{Ca}$) at the cytosolic and extracellular sides, the *l'* values may be at least five times faster than *l"*. The observed values of V'_{max} and V''_{max} for Ca^{2+}/Ca^{2+} exchange are comparable (Figure S3) as expected for the ping-pong mechanism (Figure 1E).

FIGURE S3. Mapping of NCX under conditions compatible with HDX sequence coverage of NCX_Mj. The transmembrane helices are shown above the sequence. Peptic peptides are displayed as black bars below the sequence. NCX_Mj was digested at a low pH and at a low temperature and the peptides were identified using tandem MS (40). The digestion conditions were optimized to obtain the highest sequence coverage with small or mid-size peptides (below around 30 amino acids). Pepsin, either in solution or immobilized, was tried in the presence or absence of denaturing agents. The best sequence coverage was obtained using pepsin in solution without any denaturing agent

(see Methods). The regions 41-59 (TM2) and 195-264 (from TM7 to the beginning of TM9) were covered by three and eight peptides, respectively. A small portion of TM9 was also found (peptide 259-264). Furthermore, four couples of overlapping peptides (possessing a common extremity) enabled us to locally increase the resolution, giving information on small segments (2-7 amino acids). Interestingly, the interface between the two inverted-topology repeats was covered, including the four putative ion-binding sites.

FIGURE S4: Deuterium uptake plots for peptides which are not presented in Figure 2C. Data are presented as the mean ± SD (n=3).

TABLE S1. Kinetic parameters of Na^+/Ca^{2+} and Ca^{2+}/Ca^{2+} exchange reactions.

Reaction	Observed K_m	Observed V_{max}
Ca^{2+}/Ca^{2+} exchange at varying [Ca]' and saturating [Ca]''	$K'_m = \frac{K'_{Ca} l''}{l' + l''}$	$V'_{max} = \frac{l'l''}{l' + l''}[E]_t$
Ca^{2+}/Ca^{2+} exchange at varying [Ca]'' and saturating [Ca]'	$K''_m = \frac{K''_{Ca} l'}{l' + l''}$	$V''_{max} = \frac{l'l''}{l' + l''}[E]_t$
Na^+/Ca^{2+} exchange at varying [Ca]' and saturating [Na]''	$K'_m = \frac{K'_{Ca} k''}{l' + k''}$	$V'_{max} = \frac{l'k''}{l' + k''}[E]_t$
Na^+/Ca^{2+} exchange at varying [Ca]'' and saturating [Na]'	$K''_m = \frac{K''_{Ca} k'}{k' + l''}$	$V''_{max} = \frac{k'l''}{k' + l''}[E]_t$

The initial rates of Na^+/Ca^{2+} and Ca^{2+}/Ca^{2+} exchange reactions were measured by varying the concentrations of extravesicular $^{45}Ca^{2+}$ (20-2000 µM) and using saturating concentrations of intravesicular Ca^{2+} (250 µM) or Na^+ (160 mM), as described in the legend of Fig. S1 (see also Methods). The K_m and V_{max} values of the exchange reactions were derived by fitting the calculated lines to the experimental points and the k_{cat} values were calculated as $k_{cat} = V_{max}/[E]_t$, by normalizing the expression levels of $[E]_t$ using the GFP assay (see Methods). The

rate constants, l', l'', and k' represent the Ca^{2+} and Na^{+} movements across the membrane as illustrated in Fig. 1E. The K_d value represents the observed equilibrium constant for Ca^{2+} binding in the isolated preparation of NCX_Mj. The initial rates of Na^{+}/Ca^{2+} and Ca^{2+}/Ca^{2+} exchange reactions were measured by varying the concentrations of extravesicular $^{45}Ca^{2+}$ (20-2000 µM) and using saturating concentrations of intravesicular Ca^{2+} (250 µM) or Na^{+} (160 mM), as described in the legend of Fig. S1 (see also Methods). The K_m and V_{max} values of the exchange reactions were derived by fitting the calculated lines to the experimental points and the k_{cat} values were calculated as $k_{cat} = V_{max}/[E]_t$, by normalizing the expression levels of $[E]_t$ using the GFP assay (see Methods). The rate constants, l', l'', and k' represent the Ca^{2+} and Na^{+} movements across the membrane as illustrated in Fig. 1E. The K_d value represents the observed equilibrium constant for Ca^{2+} binding in the isolated preparation of NCX_Mj.

REEFERENCES

1. Giladi, M., et al. Ca^{2+}-driven interdomain switch of NCX: Structural and biochemical studies of the two-domain Ca^{2+} sensor. *PloS One* **7,** e39985 (2012).
2. Boyman, L., Mikhasenko, H., Hiller, R. & Khananshvili D. Kinetic and equilibrium properties of regulatory calcium sensors of NCX1 protein. *J. Biol. Chem.* **284**, 6185-6193 (2009).
3. Almagor, L., et al. Functional asymmetry of bidirectional Ca^{2+}-movements in an archaeal sodium-calcium exchanger (NCX_Mj). *Cell calcium* **56**, 276-284 (2014).
4. Khananshvili, D., Weil-Maslansky, E. & Baazov, D. Kinetics and mechanism: modulation of ion transport in the cardiac sarcolemma sodium-calcium exchanger by protons, monovalent, ions, and temperature. *Ann. N. Y. Acad. Sci.* **779**, 217-235 (1996).

Figure S1

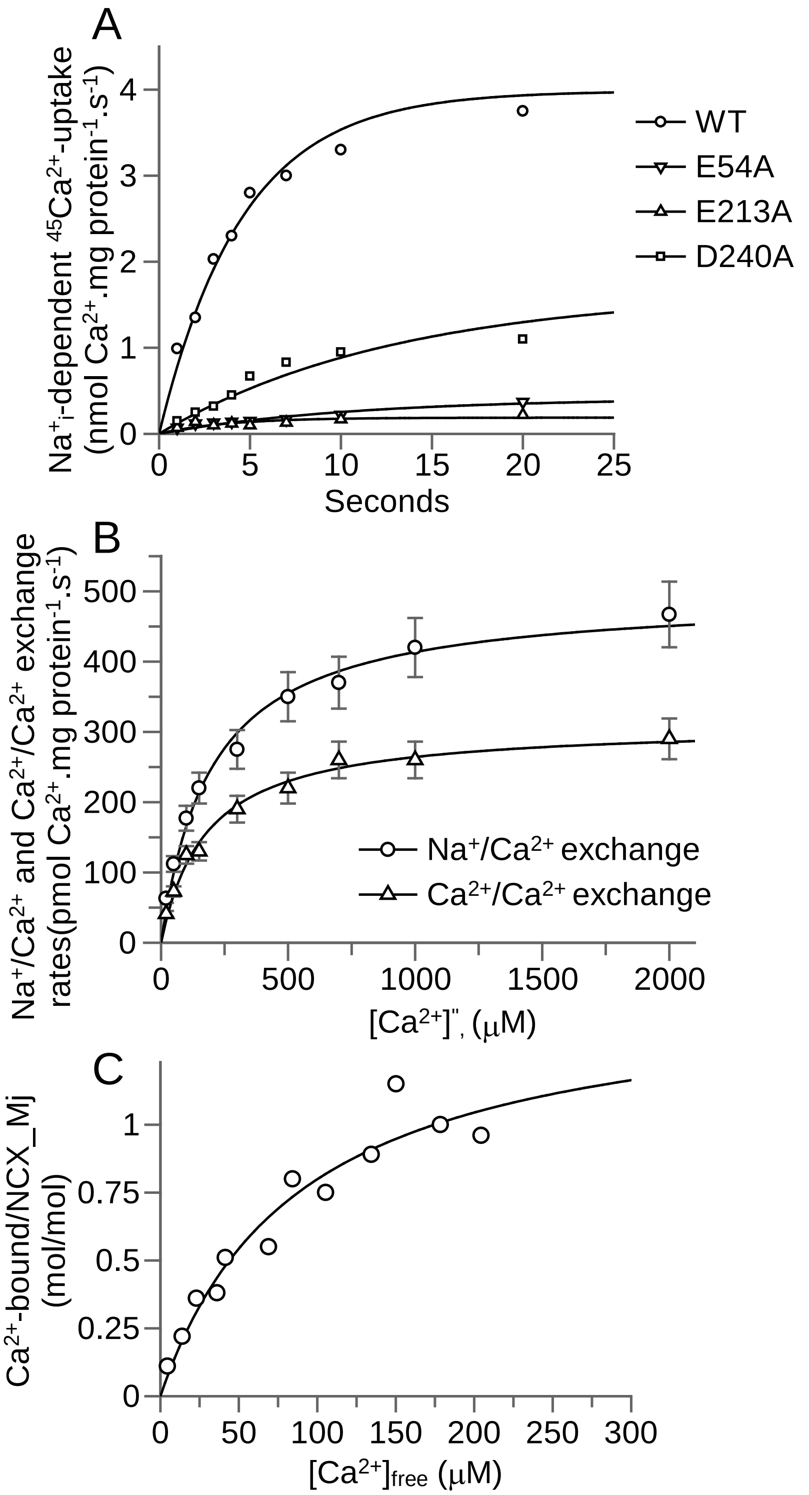
A
Na+i-dependent 45Ca2+-uptake (nmol Ca2+.mg protein-1.s-1)
Seconds
WT
E54A
E213A
D240A
B
Na+/Ca2+ and Ca2+/Ca2+ exchange rates(pmol Ca2+.mg protein-1.s-1)
[Ca2+]", (μM)
Na+/Ca2+ exchange
Ca2+/Ca2+ exchange
C
Ca2+-bound/NCX_Mj (mol/mol)
[Ca2+]free (μM)

Figure S2

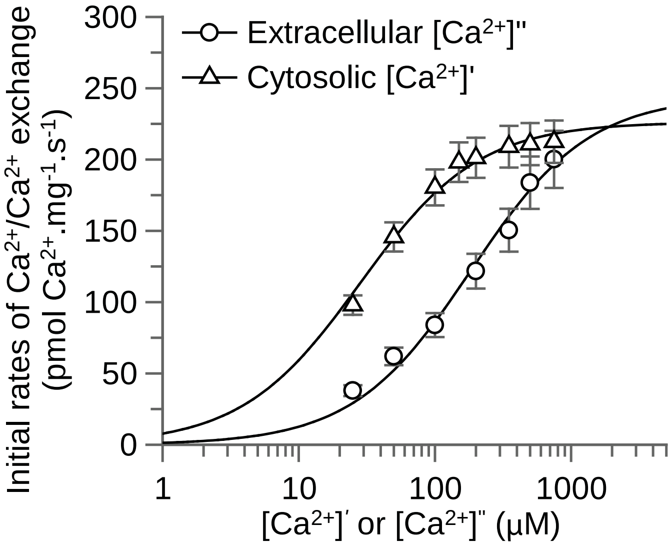

Extracellular [Ca2+]"
Cytosolic [Ca2+]'
Initial rates of Ca2+/Ca2+ exchange
(pmol Ca2+.mg-1.s-1)
300
250
200
150
100
50
0
1
10
100
1000
[Ca2+]' or [Ca2+]" (µM)

Figure S3

```
                TM1                                  TM2A       TM2B       TM2C
1   MVILGVGYFL LGLILLYYGS DWFVLGSERI ARHFNVSNFV IGATVMAIGT SLPEILTSAY 60

TM2C          TM3A              TM3B                     TM4
61  ASYMHAPGIS IGNAIGSCIC NIGLVLGLSA IISPIIVDKN LQKNILVYLL FVIFAAVIGI 120

             TM5                                         TM6
121 DGFSWIDGVV LLILFIIYLR WTVKNGSAEI EENNDKNNPS VVFSLVLLII GLIGVLVGAE 180

      TM6               TM7A    TM7B        TM7C                TM8A
181 LFVDGAKKIA LALDISDKVI GFTLVAFGTS LPELMVSLAA AKRNLGGMVL GNVIGSNIAD 240

       TM8B                     TM9                        TM10
241 IGGALAVGSL FMHLPAENVQ MAVLVIMSLL LYLFAKYSKI GRWQGILFLA LYIIAIASLR 300

301 MGGGSENLYF Q 311
```

Figure S4

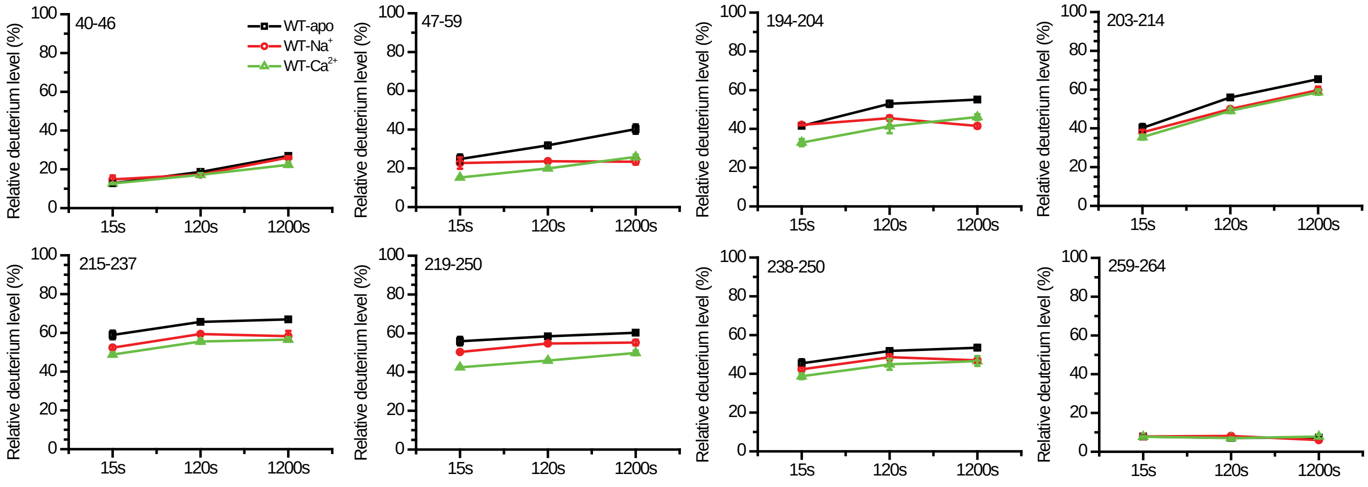

40-46
47-59
194-204
203-214
215-237
219-250
238-250
259-264
WT-apo
WT-Na+
WT-Ca2+
Relative deuterium level (%)
15s
120s
1200s
